# Progress in Surgery

**Published:** 1894-11-10

**Authors:** 


### Progress in Surgery.

### SURGERY OF THE INTESTINES.

**Intestinal Approximation.**—Dr. Murphy gives[1] brief histories of thirty-two cases of intestinal approximation with the use of the Murphy button, and forms the following conclusions: (1) The more rapidly the operation is performed the less the danger from shock. (2) The less the manipulation and exposure of the intestine, the less the danger of infection, post-operative paralysis, and adhesions. (3) The more uniform and continuous the pressure at approximation, the greater the assurance of adhesion, and the less the liability of infiltration. (4) A line of approximation is as good as half an inch. (5) Mechanical means in the last six years have produced better results than the suture, in both lateral and end to end approximations. (6) The mortality in end to end approximation is much less than in lateral apposition, and it should always be given the preference. (7) The more perfect the juxtaposition of the various layers, the less the interposition of fibrous tissue, and the more complete the regeneration across the line of union. (8) The more extensive the approximation surface, the larger the fibrous deposit, the greater the contraction. (9) The contraction with end to end is less than with lateral approximation. (10) The juxtaposition of the similar histological layers of the wall of the intestine is an assurance against cicatricial contraction. As regards diagnosis[2], in intestinal obstruction there is no elevation of temperature, while in the early stage of perforative peritonitis there is an increased temperature. Peristalsis is greatly increased in intestinal obstruction, and continues until necrosis or peritonitis occurs, while there is a paralysis of peristalsis over the inflamed area in perforative peritonitis. He gives[3] statistical tables* of the results of lateral approximation, and gastro-enterostomy for malignant disease, which show the superiority of the "button" method.

**A New Device for Intestinal Anastomosis** is announced[4] by Dr. Byron Robinson. It consists of two metal discs, around whose circumference there is a groove. In the groove is placed a hollow rubber tube. The two discs are clamped together with a ratchet or spiral screw. The principle on which the device works is the same as Denan's cylinders. The gut is gradually necrosed through by slow elastic pressure. The rubber tube and pressure can be so controlled that the circular line of union may be of determined width. The elastic pressure avoids lateral fistula from too rapid or too violent force. The instrument is called the "rubber-faced disc."

**How much of the Intestine may be Excised** has been the subject of an experimental inquiry by Dr.

* LATERAL APPROXIMATION.

| | Cases. | Recoveries. | Deaths. | Not Given. | Mortality Percentage. |
|---|---|---|---|---|---|
| Suture | 33 | 20 | 12 | 1 | 36·4 |
| Suture with mechanical aids | 23 | 18 | 5 | — | 21·7 |
| Of these, bone plates | 14 | 11 | 3 | — | 21·4 |
| Robson's bone bobbin | 1 | 1 | — | | |
| Potato plates | 1 | — | 1 | — | 100·0 |
| Robinson's raw-hide segmented plates | 1 | — | 1 | — | 100·0 |
| Abbe rings | 4 | 1 | 3 | — | 75·0 |
| Davis mats | 1 | — | 1 | — | 100·0 |
| Mechanical means, Murphy button | 10 | 10 | | | |
| Total | 66 | 43 | 21 | 1 | 31·8 |

GASTRO-ENTEROSTOMY FOR MALIGNANT DISEASE.

| | Cases. | Recoveries. | Deaths. | Mortality per cent. |
|---|---|---|---|---|
| Method by suture | 28 | 16 | 12 | 42·8 |
| Mechanical means | 49 | 36 | 13 | 26·5 |
| Suture with mechanical aid | 8 | 1 | 7 | 87·5 |
| Total number operated | 85 | 53 | 32 | 37·6 |
| Of these the position was: | | | | |
| Side to side in | 76 | 47 | 29 | 38·2 |
| End to side | 4 | 3 | 1 | 25·0 |
| Unknown | 5 | 4 | 1 | 20·0 |
| Of the mechanical means employed: | | | | |
| Bone plates were used | 36 | 28 | 8 | 22·2 |
| Swedish turnip plates | 2 | 1 | 1 | 50·0 |
| Robinson's segmented rubber plate | 2 | 1 | 1 | 50·0 |
| Mayo Robson, bone bobbin | 2 | 1 | 1 | 50·0 |
| Murphy button | 7 | 6 | 1 | 14·3 |
| Suture with mechanical aid. Means employed: | | | | |
| Abbe catgut rings | 5 | 0 | 5 | 100 |
| Brokaw's segmented rubber rings | 2 | 1 | 1 | 50·0 |
| Elastic ligatures | 1 | 0 | 1 | 100 |

Trzebicky. From the point of view of practical surgery on the human subject, he thinks[5] himself justified in deducing from his experiments (on dogs) that half the jejuno-ileum—*i.e.*, at least 280 centimetres, or about 9 feet—may be removed without running unjustifiable risk, provided that no complications are present.

**The Artificial Production of Reversed Peristalsis** (for determining the direction in which the intestine runs) by means of carbonate of sodium is said[6] by R. T. Morris to be dangerous, as it is apt to produce so violent a contraction as to produce an intussusception. He believes chloride of sodium to be harmless for this purpose, but it is as well to remove the salt as soon as its object has been accomplished.

**Resection of Gangrenous Bowel.**—Mr. Lockwood quotes[7] a case of strangulated hernia of eighty-one hours' duration, in which the gut had become gangrenous. Believing that resection and immediate suture is less dangerous than the formation of an artificial anus with subsequent resection, he performed the former operation, removing about four inches of gut, and uniting the ends by Czerny-Lembert's method without any special apparatus, and the patient made an uninterrupted recovery. His reasons for deciding against the formation of an artificial anus were that it exposed the patient to (1) non-relief of the obstruction; (2) spread of the gangrene or ulceration; (3) spread of the septic peritonitis; (4) death from inanition; (5) a further operation for the close of the artificial anus. H. H. Clutton thinks[8] it would perhaps be better to do a secondary resection a few days after the first operation. If we wait two or three weeks before the secondary resection the bowel has contracted adhesions, and the two ends no longer correspond in size, so that the operation may thus be more difficult. If, on the other hand, secondary resection is undertaken in a few days after the formation of an artificial anus, adhesions will not be so troublesome, the doubtful area to excise will be defined, the two ends of the bowel will more readily fit, and the patient better able than at the first operation to stand what is necessary to be done. In some cases it might be desirable to remove all the damaged bowel at the first operation, and having brought the two ends with the mesentery up into the wound complete the operation in a few days by reunion of the divided intestines.

[1] Chicago Clinical Review, June, 1894. [2] Medical Record, June 2, 1894. [3] Ibidem, May 26 and June 2, 1894. [4] Medical Record, June 9, 1894, p. 726. [5] Lancet, August 4, 1894, p. 265. [6] Medical Record, March 31, 1894, p. 389. [7] Brit. Med. Journ., March 17, 1894, p. 577. [8] Annals of Surgery, April, 1894, p. 416.

*(To be continued.)*

